# Observing Interactions between Children and Adolescents and their Parents: The Effects of Anxiety Disorder and Age

**DOI:** 10.1007/s10802-015-0005-z

**Published:** 2015-03-20

**Authors:** Polly Waite, Cathy Creswell

**Affiliations:** School of Psychology and Clinical Language Sciences, University of Reading, Whiteknights, Reading, RG6 6AL UK

**Keywords:** Childhood, Adolescence, Anxiety, Parenting, Behavior

## Abstract

Parental behaviors, most notably overcontrol, lack of warmth and expressed anxiety, have been implicated in models of the development and maintenance of anxiety disorders in children and young people. Theories of normative development have proposed that different parental responses are required to support emotional development in childhood and adolescence, yet age has not typically been taken into account in studies of parenting and anxiety disorders. In order to identify whether associations between anxiety disorder status and parenting differ in children and adolescents, we compared observed behaviors of parents of children (7–10 years) and adolescents (13–16 years) with and without anxiety disorders (*n* = 120), while they undertook a series of mildly anxiety-provoking tasks. Parents of adolescents showed significantly lower levels of expressed anxiety, intrusiveness and warm engagement than parents of children. Furthermore, offspring age moderated the association between anxiety disorder status and parenting behaviors. Specifically, parents of adolescents with anxiety disorders showed higher intrusiveness and lower warm engagement than parents of non-anxious adolescents. A similar relationship between these parenting behaviors and anxiety disorder status was not observed among parents of children. The findings suggest that theoretical accounts of the role of parental behaviors in anxiety disorders in children and adolescents should distinguish between these different developmental periods. Further experimental research to establish causality, however, would be required before committing additional resources to targeting parenting factors within treatment.

Anxiety disorders are highly prevalent among children and adolescents (Essau and Gabbidon 2013) and have negative consequences (Last et al. 1997; Pine et al. 1998). As such it is critical to identify key factors that are involved in the development and maintenance of anxiety disorders in young people in order to inform prevention and treatment. Theoretical models have implicated a number of family factors that appear to play a role, including genetics, adverse life events, parental psychopathology, as well as parenting behaviors (Creswell et al. 2011; Rapee et al. 2009).

Parental behaviors, most notably overcontrol, lack of warmth and expressed anxiety are hypothesized to promote anxiety among children and young people, especially among those who already experience elevated trait anxiety (Wood et al. 2003). Overcontrol is characterized by parental over-involvement, where the parent takes over doing tasks that the child is capable of doing independently and encourages the child to be excessively dependent on them, in an attempt to protect the child from possible distress or harm (e.g., McLeod et al. 2007; Rapee 1997; Rothbaum and Weisz 1994; Wood 2006). Theoretical models propose that parental overcontrol impacts on the child’s sense of self-efficacy, limits his or her experience of novel situations and constrains his or her ability to manipulate or engage in the environment independently (Chorpita and Barlow 1998; Rapee 1997; Wood 2006). In contrast, parental autonomy-granting, where the child is encouraged to be independent, develop his or her own opinions and make decisions for himself or herself, has been suggested to increase a sense of mastery over the environment, leading to a reduction in anxiety (Chorpita and Barlow 1998). Two meta-analyses, including studies of both community and clinical participants, have found a medium-sized association between parental control and child anxiety (McLeod et al. 2007; van der Bruggen et al. 2008), with relatively stronger associations for the sub-dimension of autonomy granting than other dimensions, such as over-involvement, particularly when using observational assessment strategies (McLeod et al. 2007).

A further dimension of potential relevance is that of rejection, where the parent may be critical or hostile towards the child, or the relationship is characterized by a lack of warmth, involvement, emotional support or reciprocity (McLeod et al. 2007). This may increase the child’s sensitivity to anxiety by undermining his or her ability to regulate emotion (Chorpita and Barlow 1998; McLeod et al. 2007). In their meta-analysis, McLeod et al. (2007) reported a small but significant association between parental rejection and child anxiety; although there is some need for caution as many of the studies assessed parental rejection on the basis of child/adolescent report which may be subject to bias. Furthermore it has been suggested that parental rejection or lack of warmth may be more strongly associated with symptoms of depression than anxiety (Rapee 1997), making it possible that associations with anxiety may actually be accounted for by overlapping symptoms of low mood.

In addition to the two broad constructs of control and rejection, it has been hypothesized that parents may reinforce child anxiety by modeling and/or reinforcing anxious behaviors (Rachman 1977), through ‘anxious rearing’ behaviors. There is some evidence that parental expressed anxiety promotes the development of anxious or fearful cognitions, behaviors and symptoms (Askew and Field 2007; De Rosnay et al. 2006; Gerull and Rapee 2002; Grüner et al. 1999; Waters et al. 2012).

Although there is now a large body of research examining these parenting behaviors in relation to anxiety in young people, it is striking that age has not typically been taken in to account as theories of normative development have proposed that different parental responses are required to support emotional development in childhood and adolescence. One of the central tasks in adolescence is for the adolescent to separate from parents and become increasingly independent as they approach adulthood (Steinberg 2001). As such, there are greater expectations on the adolescent to be autonomous, especially within the school environment (Eccles and Harold 1993). This then requires a renegotiation of the parent–child relationship and for parents to find an effective balance between autonomy and control (Steinberg and Silk 2002). Additionally, normative changes in adolescence have implications for parental warmth/rejection. Larson et al. (2002) examined negative affect in children and adolescents from the age of 10 to 14 years and found that as age increased, so did reports of daily negative emotional states. As cognitive abilities develop, adolescents’ more critical, logical thinking results in parents no longer being idealized and previously accepted parental rules being challenged (Steinberg and Silk 2002). Although there do not appear to be higher rates of conflict with parents in adolescence generally (Steinberg 2001), affect intensity during conflict has been shown to increase from early to mid-adolescence (Laursen et al. 1998) and adolescents report decreasing rates of affectionate behavior towards their parents (Eberly and Montemayor 1999). Compared to children, adolescents undertake fewer shared activities with their parents and spend considerably less time with their family (Larson and Richards 1991). Taken together, these results suggest that parenting of anxious adolescents may involve lower levels of warmth and higher levels of rejection/hostility than middle childhood, and that parental control may continue to be of relevance. However, this has not been addressed in the existing literature. Instead the majority of studies involve pre-adolescent children (e.g., Grüner et al. 1999; Hirshfeld et al. 1997; Siqueland et al. 1996), or include both children and adolescents with analyses conducted across the age ranges (e.g., Barrett et al. 2002; Muris et al. 1996).

A recent systematic review examining evidence for an association between parenting behaviors and adolescent anxiety (Waite et al. 2014) found fairly consistent, preliminary evidence for an association between anxiety and perceived parental control and anxious rearing in adolescence, with effect sizes in the small to medium range (e.g., van Brakel et al. 2006; Van Zalk and Kerr 2011; Wijsbroek et al. 2011; Wolfradt et al. 2003). The findings relating to an association between adolescent anxiety and perceived parental rejection and lack of warmth were somewhat less consistent, but where associations were significant, effect sizes were also in the small to medium range (e.g., Hudson and Rapee 2001; Schwartz et al. 2012; Verhoeven et al. 2012). The results of the two studies that have examined prospective relationships between adolescent anxiety and perceptions of parental control (Van Zalk and Kerr 2011; Wijsbroek et al. 2011) provided support for bi-directional effects although interestingly, effect sizes were somewhat stronger (with small to medium effects) for adolescent symptoms of anxiety predicting later higher levels of perceived parental control than for perceptions of control predicting later adolescent anxiety. This is further supported by the findings of Hale et al. (2013), where adolescent symptoms of generalized anxiety disorder predicted later perceptions of both parental rejection and overcontrol. However, the majority of the studies identified were limited by a reliance on adolescent reported parenting and restriction to community populations, limiting conclusions that can be drawn about actual (rather than perceived) parental responses and clinical groups.

Only one study to date has examined associations between parenting and anxiety separately for children and adolescents (Hudson and Rapee 2001). Post-hoc analyses following an observational study with clinically anxious and non-clinical children, identified a significant effect of child/adolescent age (age groups were 7–9, 10–11 and 12–13 years) on observed maternal involvement during two cognitive tasks, with mothers providing significantly less help as the child got older. There was not, however, a significant interaction between child/adolescent age and anxiety status on maternal involvement, nor a significant effect of age, or the interaction between age and anxiety status, for maternal negativity. While these findings highlight the potential differences in parenting behaviors with child age, conclusions are limited by (i) the small sample sizes within each subgroup (clinically anxious *n* = 43 and non-clinical *n* = 32, split between three age groups), and (ii) the broad parenting constructs used, which included, for example, consideration of aspects of behaviors (such as parental positioning) which could reflect parental encouragement in the control scale, and behaviors which could reflect maternal anxiety (such as maternal tension) in the negativity scale. This is an important consideration given suggestions that more specifically defined parenting behaviors are more strongly associated with child anxiety disorder status (McLeod et al. 2007). As such, further research is necessary to help identify the critical parental processes that are associated with anxiety disorders during different developmental periods to help inform clinical interventions targeted at specific age ranges.

The current study builds on previous work by using observational methods and examining the effects of anxiety disorder, age group and their interaction on parenting behaviors. As parental responses are likely to be influenced by the degree to which offspring express anxiety during interaction tasks (Creswell et al. 2013; Rapee 1997), we also measured child/adolescent observed behaviors and accounted for this in analyses. The following hypotheses were examined:

1. Parents of offspring with anxiety disorders will exhibit significantly higher levels of intrusiveness and anxiety and significantly lower levels of positive behaviors (i.e. warmth, engagement and encouragement) than parents of non-anxious offspring.
2. Parents of children will show significantly higher levels of intrusiveness and positive behaviors (i.e. warmth, engagement and encouragement) than parents of adolescents.

Given the lack of theory or prior evidence to guide directional hypotheses we also set out to explore whether offspring age group moderated the association between anxiety disorder status and parenting behaviors.

## Method

### Participants

Ethical approval for the study was given by the National Research Ethics Service (NRES) London - Brent Research Ethics Committee and the University of Reading Ethics Committee. All participants provided informed consent prior to taking part in the research.

#### Children and Adolescents with Anxiety Disorders

All children and adolescents with anxiety disorders were referred by primary and secondary care services for the assessment and treatment of an anxiety disorder. To be included in the study, all children/adolescents were required to meet diagnostic criteria for a current anxiety disorder on the Anxiety Disorders Interview Schedule (ADIS-C/P; Silverman and Albano 1996) and for this to be identified as the primary problem. They were not invited to participate if they had psychotic symptoms, substance dependence, an autistic spectrum disorder, conduct disorder, a risk of deliberate self-harm, if they were taking psychoactive medication, currently receiving therapy for their anxiety disorder or if they, or their parent, did not understand and speak English at a level that would enable them to complete the procedures or had any significant intellectual impairment. Five adolescents were excluded based on the study exclusion criteria (two because of a risk of deliberate self-harm and three because they were taking psychoactive medication). No children were excluded on the basis of the study exclusion criteria.

Thirty adolescents aged between 13 and 16 years were recruited prior to commencing treatment, along with the parent identified as their primary caregiver. We then selected 30 children aged 7–10 years, who had been diagnosed with an anxiety disorder and had completed the same assessment with their mothers as part of a wider study. The children with anxiety disorders were selected to match the adolescent group on their primary anxiety disorder, comorbid mood and behavior disorders, gender, ethnicity and socio-economic status. Table 1 provides demographic information for all participants. As shown in Table 1, although the adolescent group included parents of both sex, very few fathers took part and so the difference between the groups was not significant.

**Table 1:** Sample characteristics

	Anxious children (*n* = 30)	Non-anxious children (*n* = 30)	Anxious adolescents (*n* = 30)	Non-anxious adolescents (*n* = 30)	Statistics
Child/adolescent gender (boys: girls)	14:16	20:10	14:16	16:14	*χ* ^*2*^(3) = 3.21, *p* = .36
Age in months (mean, *SD*, range)	112.20 (10.49), 94–130 ^a^	110.60 (9.77), 96–131	181.50 (13.48), 158–198 ^a^	183.03 (13.79), 161–205	*F*(3, 116) = 348.21, *p* < .001
Ethnicity (% White British)	93 %	93 %	93 %	90 %	*χ* ^*2*^(3) = 15.03, *p* = .95
Family SES (% “higher” or “professional”)	67 %	73 %	67 % ^a^	97 % ^a^	*χ* ^*2*^(3) = 10.01, *p* = .02
Parent gender (% female)	100 %	100 %	93 %	90 %	*χ* ^*2*^(3) = 5.64, *p* = .13
SCAS-c total (mean, *SD*)	36.20 (19.03)	27.89 (10.74)	39.23 (17.62) ^b^	10.97 (5.54) ^b^	*F*(3, 111) = 22.30, *p* < .001
SCAS-p total (mean, *SD*)	36.03 (14.75) ^a^	13.97 (5.86) ^a^	31.77 (18.52) ^b^	6.87 (3.15) ^b^	*F*(3, 111) = 36.32, *p* < .001
SMFQ-c total (mean, *SD*)	6.70 (4.50)	4.79 (3.20)	7.34 (5.77) ^b^	2.17 (2.41) ^b^	*F*(3, 111) = 8.86, *p* < .001
SMFQ-p total (mean, *SD*)	6.60 (4.97) ^a^	1.83 (2.28) ^a^	8.63 (7.89) ^b^	1.43 (1.92) ^b^	*F*(3, 111) = 15.01, *p* < .001

Where self-report data was missing, this was less than 10 % of the dataset. Superscript letters refer to pairwise comparisons (conducted for children with AD versus adolescents with AD, children with AD versus non-anxious children, and adolescents with AD versus non-anxious adolescents); means that share subscripts within rows are significantly different at *p* < .05

For both groups, the primary anxiety disorder diagnoses were: social anxiety disorder (*n* = 8, 27 %), specific phobia (*n* = 9, 30 %), generalized anxiety disorder (*n* = 7, 23 %), panic disorder with/without agoraphobia (*n* = 5, 17 %), and agoraphobia without panic disorder (*n* = 1, 3 %). The groups did not differ significantly in the mean severity rating for the primary diagnosis (children: mean = 5.30 (*SD* = 0.84); adolescents: mean = 5.53 (*SD* = 0.94); *t*(58) = 1.02, *p* = .31). The children did, however, experience significantly more comorbid anxiety disorders than the adolescents (children: mean = 1.3 (*SD* = 1.21); adolescents: mean = 0.77 (*SD* = 0.82); *t*(58) = −2.00, *p* = .05). In terms of comorbid mood disorders, 4 young people (13 %) in each group had been diagnosed with dysthymic disorder and one young person (3 %) with major depressive disorder. For comorbid behavior disorders, 2 young people (7 %) in each group were diagnosed with oppositional defiant disorder. As can be seen in Table 1, the clinical groups did not differ significantly on self- and parent-report measures of symptoms of anxiety (Spence Child Anxiety Scale - Child and Parent versions (SCAS-C/P); Spence 1998) (SCAS-C: *t*(57) = 0.48, *p* = .65; SCAS-P: *t*(58) = −0.99, *p* = .35), self- and parent-report measures of symptoms of low mood (Short Mood and Feelings Questionnaire - Child and Parent versions (SMFQ-C/P); Angold et al. 1995) (SMFQ-C: *t*(57) = 0.48, *p* = .63; SMFQ-P: *t*(57) = 1.25, *p* = .25) and parent-reported behavioral problems (Strengths and Difficulties Questionnaire (SDQ-P) conduct subscale; Goodman 1997) (SDQ-P conduct: *t*(58) = −1.19, *p* = .24).

#### Non-anxious Children and Adolescents

Thirty non-anxious adolescents aged 13–16 years were recruited, along with their primary caregiver. A further 30 non-anxious children aged 7–10 years were selected from a wider study to match the children/adolescent groups where possible on demographic variables. All non-anxious participants were recruited through advertisements in newsletters of local schools and youth groups. Families received a gift voucher as a token of appreciation for their participation. To be included in the study, all non-anxious participants were required to score below clinical cut-offs on the SCAS-P and the SMFQ-P and the parent identified as their primary caregiver also had to agree to take part. As with the anxious participants, non-anxious children and adolescents were not eligible if they, or their parent, did not understand and speak English at a level required to participate in the study, had any significant intellectual impairment, or if they were having therapy or taking medication for any psychological problems. As can be seen in Table 1, there was not a significant difference in age between the two child groups and the two adolescent groups, nor were there any significant differences between all the groups for ethnicity or parent gender. However, significantly more of the non-anxious adolescent group came from families where parental occupational status was classified as higher/professional (Office for National Statistics 2005) than the other groups.

As expected, on symptom measures, the adolescents with anxiety disorders scored significantly higher than the non-anxious adolescents on self- and parent-report measures of symptoms of anxiety (SCAS-C: *t*(56) = 8.18, *p* = .001; SCAS-P: *t*(56) = 7.00, *p* = .001 ), low mood (SMFQ-C: *t*(56) = 4.44, *p* < .01; SMFQ-P: *t*(56) = 4.83, *p* < .01), and parent-reported behavioral problems (SDQ-P conduct: *t*(56) = 2.38, *p* < .05). Similarly, the children with anxiety disorders scored significantly higher than the non-anxious children on parent-reported symptoms of anxiety (SCAS-P: *t*(55) = 7.36, *p* = .001), low mood (SMFQ-P: *t*(55) = 4.49, *p* = .001), and behavioral problems (SDQ-P conduct: *t*(55) = 2.85, *p* < .01). Although the children with anxiety disorders reported a greater number of symptoms of anxiety and low mood than the non-anxious children, the differences fell just short of significance (SCAS-C: *t*(55) = 1.96, *p* = .07); SMFQ-C: *t*(55) = 1.80, *p* = .08).

### Procedure

For the children and adolescents with anxiety disorders, the child/adolescent and their parent were seen separately by trained psychology BSc/MSc graduates (assistant psychologists or trainee clinical psychologists) to undertake a diagnostic assessment (relating to the child/adolescent) and complete standardized questionnaires. For the non-anxious children and adolescents, if they expressed an interest in the study, they were sent consent forms, information sheets and the screening measures to complete and return. Potential participants from all groups were then contacted by a researcher to discuss further, and if they were eligible and agreed to take part, to arrange the assessment appointment. This appointment involved an observational assessment at the university, during which they carried out a series of mildly anxiety-provoking tasks, which were video-recorded. The procedure was administered by the researcher (PW) or trained psychology (BSc/ MSc) graduates who received regular supervision. Videos of the parent-offspring interactions were coded by trained psychology (BSc/ MSc) graduates who were blind to both participant group and the study hypotheses.

### Measures

#### Diagnoses

Children and adolescents’ diagnoses were determined using the ADIS-C/P (Silverman and Albano 1996). This is a structured interview, with good psychometric properties (Silverman et al. 2001), designed to assess current DSM-IV anxiety disorders, as well as current mood and behavioral disorders. As is standard, if the child/adolescent met symptom criteria for a diagnosis, on the basis of his/her report or that of his/her parent, the assessor assigned a Clinician Severity Rating (CSR), ranging from 0 (absent or none) to 8 (very severely disturbing/disabling); a CSR of 4 or more based on the child/adolescent and/or parent report indicated the child/adolescent met criteria for diagnosis. The diagnosis with the highest CSR was classed as the primary diagnosis. For each assessor, the first 20 interviews were discussed with a consensus team led by an experienced diagnostician (Consultant Clinical Psychologist). After 20 ADIS assessments had been double coded by the consensus team, reliability was formally checked and raters were required to be reliable at a kappa/intraclass correlation of 0.85 before being considered reliable. Once reliability had been achieved, every sixth independent assessment was discussed with the consensus team to prevent rater drift. Overall reliability for the assessment team was good to excellent; reliability for the ADIS-C/P diagnosis was: child report, *M* = 0.97 (range 0.88 – 1.00), parent report, *M* = 0.98 (range 0.92 – 1.00) and for CSR scores was: child report, *M* = 0.98 (range 0.91 – 1.00) and parent report, *M* = 0.98 (range 0.96 – 1.00).

#### Symptom Measures

The Spence Children’s Anxiety Scale (SCAS-C/P; Spence 1998) assesses child/adolescent and parent-reported anxiety symptoms. It includes 38 items (and 6 positive filler items in the child version), each scored on a 4-point Likert scale, ranging from 0 (never) to 3 (always). The measure has been validated for use with children/adolescents aged from 6 to 18 years and both versions have good reliability, as well as discriminant and convergent validity (Nauta et al. 2004; Spence et al. 2003). Internal consistency for these scales was excellent (SCAS-C *α* = 0.92; SCAS-P *α* = 0.94).

The Short Mood and Feelings Questionnaire (SMFQ-C/P; Angold et al. 1995) is a self-report measure to assess child/adolescent depressive symptoms. There are versions for children/adolescents and parents to complete; both versions have 13 items and each item is scored on a 3-point scale (*not true*, *sometimes* or *true*). The scale has been validated with children/adolescents aged 6–17 years and has good internal reliability and discriminant validity (Angold et al. 1995). Internal consistency for the SMFQ was good to excellent (SMFQ-C *α* = 0.86; SMFQ-P *α* = 0.93).

The conduct problems subscale of the Strengths and Difficulties Questionnaire (SDQ-P) (Goodman 1997) was administered to assess parent-reported behavioral disturbance. Five items are scored on a 3-point scale (*not true*, *somewhat true* and *certainly true*). The scales show acceptable internal consistencies and retest reliability (Goodman 2001). The parent-report version of the SDQ was used as parents are often considered to be most reliable in reporting on children’s externalizing symptoms (Grills and Ollendick 2003). Although internal consistency was poor (SDQ-P conduct problems *α* = 0.57), this is likely to reflect the relatively low number of items in the subscale.

#### Observational Measures of Parenting

Three challenge tasks were administered to participants: a mysterious black box, tangram puzzles and a speech task. These tasks have been demonstrated to be associated with mild levels of self-reported and observed anxiety and increases in autonomic arousal, in comparison to baseline, for both children with anxiety disorders and non-anxious children (Alkozei et al. 2015). The black box task was designed to invoke mild anxiety around specific objects following Creswell et al. (2013). Children and parents were first asked to discuss the possible contents of the box before the child/adolescent placed his/her hands through each of four holes (with the contents obscured) to discover what was inside. The box contained a fluffy toy, a rubber toy, a feather boa and some slime. The tangram task was designed to invoke anxiety around performance following Hudson and Rapee (2001). The child/adolescent was instructed to put puzzle pieces together to fit into larger shape templates within 5 min. Following Hudson and Rapee (2001), the puzzles were selected to be difficult. Parents were told that this was a test of their child’s ability and given the puzzle solutions, but were told to help their child only if they needed it. The speech task followed the procedures of Creswell et al. (2013) and Gar and Hudson (2008). The child/adolescent and parent were given some suggestions of topics to talk about and were left alone for between 3 and 5 min to prepare for the presentation. The parent was told that most children/adolescents found it a bit difficult to get going, so they could help their son or daughter if they thought they really needed it. In the second part, the parent was asked to introduce the presentation and then the child/adolescent was given between 3 and 5 min to present their speech, in the presence of their parent. The tangram and the speech task were adapted for the different age groups in terms of level of difficulty of the puzzles and the length of time given for the speech task, but the mysterious box task was identical for both age groups because the task involved dealing with the unknown and therefore adaptations were unnecessary.

Observed child/adolescent and parental behaviors were rated by psychology graduates using a coding scheme developed by Murray et al. (2012) and adapted for this age range and tasks by Creswell et al. (2013). Each behavior was rated on a scale of 1 to 5, with 1 being *none* and 5 *pervasive/strong*. Each minute was rated separately and then a mean score was calculated for each behavior on each task. For parenting behaviors, the following codes were used:

Negative behaviors, each rated each minute along the 1 (*none*) to 5 (*pervasive/strong*) scale:

- Expressed anxiety (i.e., modeling of anxiety). Anxiety in facial expression (e.g., fearful expression, biting lip), body movements (e.g., rigid posture, wringing hands), and speech (e.g., rapid, nervous, or inhibited).
- Passivity. Withdrawn and inhibited, unresponsive to child behavior and communication (e.g., physically distant, silent).
- Promotion of avoidance. Actively encourages/supports child avoidance of task (e.g., saying “you don’t have to do it”).
- Overprotection. Initiates emotional and/or practical support that is not required (e.g., stroking/ kissing/offering unnecessary help while child/adolescent manages independently).
- Intrusiveness. Interferes, verbally or physically, cutting across child behavior, attempts to take over and imposes own agenda.

Positive behaviors, each rated each minute along the 1 (*none*) to 5 (*pervasive/strong*) scale:

- Encouragement (autonomy-promotion). Provides positive motivation to child to engage in the task, showing both interest in the task (e.g., making suggestions and asking questions) and enthusiasm regarding both task and child capacity/efforts (e.g., displaying positive affect, positive tone of voice, smiling, laughing).
- Warmth. Affectionate, expresses positive regard for child/adolescent, both verbally and physically.
- Engagement. Involvement and interest in what the child is doing (e.g., orienting body to child, asking the child what they are doing, showing an interested response).

For child/adolescent behavior, the following codes were used, each rated each minute along the 1 (*none*) to 5 (*pervasive/strong*) scale:

- Expressed anxiety. Anxiety in facial expression (e.g., fearful expression, biting lip), body movements (e.g., rigid posture, wringing hands), and speech (e.g., rapid, nervous, inhibited).
- Avoidance. Non-verbal or verbal avoidance (e.g., reluctance or refusal to approach or do the task).

Coders were trained using video tapes that were not part of this study. For each task, a second coder independently rated a random sample of 20 videos and reliability was formally checked. Coders were required to be reliable at a kappa/intraclass correlation of 0.7 or above for every code before being considered reliable. Intraclass correlations showed high levels of agreement between raters for all codes: parental expressed anxiety, *M* = 0.95 (range 0.88 – 1.00 across tasks/raters); passivity, *M* = 0.97 (range 0.95 – 1.00); promotion of avoidance, *M* = 0.97 (range 0.85 – 1.00); overprotection, *M* = 0.96 (range 0.87 – 1.00); intrusiveness, *M* = 0.93 (range 0.78 – 0.99); encouragement, *M* = 0.93 (range 0.85 – 0.98); warmth, *M* = 0.96 (range 0.93 – 0.98); engagement, *M* = 0.90 (range 0.78 – 0.98); child/adolescent expressed anxiety, *M* = 0.93 (range 0.91–0.94), and child/adolescent avoidance, *M* = 0.98 (range 0.92 – 1.00).

## Results

### Data Reduction, Analytic Strategy and Preliminary Analyses

As the majority of continuous data was highly skewed and violated assumptions of normality, analyses were run parametrically with 1,000 bootstrap samples. Overprotection, passivity and promotion of avoidance were uncommon, with only 5.2–12.0 % of parents rated above the minimum score across all tasks for these behaviors, and therefore these codes were not included in the analyses. The codes of warmth and engagement correlated highly on every task (*r* = 0.60 – 0.85) and were therefore combined for analyses as a ‘warm engagement’ dimension. Inter-correlations between parenting behaviors across all tasks are shown in Table 2. Child/adolescent observed anxiety and avoidance correlated at 0.50 and so analyses were run for these behaviors separately and then as a single, combined variable. As the results were largely consistent when the variables were combined, for brevity, this will be presented. We examined behaviors in each different task and across all tasks combined; again, as findings were broadly consistent, we have presented the combined behavior ratings across all tasks for brevity (see Table 3).

**Table 2:** Spearman’s correlations between different parenting behavior codes

	Expressed anxiety	Passivity	Promotion of avoidance	Overprotection	Intrusiveness	Encouragement	Warmth	Engagement
Expressed anxiety	–							
Passivity	0.28**	–						
Promotion of avoidance	0.17	−0.03	–					
Overprotection	0.15	0.03	−0.01	–				
Intrusiveness	0.53***	0.15	0.18*	0.04	–			
Encouragement	−0.15	−0.39**	−0.02	0.18	−0.19*	–		
Warmth	0.06	−0.06	−0.10	0.21*	−0.12	0.58***	–	
Engagement	0.12	−0.16	−0.08	0.22*	0.07	0.59***	0.79***	–

* *p* < .05, ** *p* < .01, *** *p* < .001

**Table 3:** Group differences in child/adolescent and parent behaviors across all tasks

		Anxious children (*n* = 30)	Non-anxious children (*n* = 30)	Anxious adolescents (*n* = 30)	Non-anxious adolescents (*n* = 30)
Child/adolescent	Anxiety/avoidance (mean, *SD*)	1.50 (0.32)	1.45 (0.24)	1.64 (0.39) ^a^	1.23 (0.20) ^a^
Parent	Anxiety (mean, *SD*)	1.93 (0.56) ^a^	1.80 (0.39)	1.23 (0.16) ^a^ ^b^	1.15 (0.15) ^b^
	Intrusiveness (mean, *SD*)	1.65 (0.57) ^a^	1.82 (0.44)	1.31 (0.22) ^a^ ^b^	1.19 (0.19) ^b^
	Positive behaviour (mean, *SD*)	3.30 (0.44) ^a^	3.12 (0.49)	2.80 (0.36) ^a^ ^b^	3.13 (0.38) ^b^
	Encouragement (mean, *SD*)	2.88 (0.53)	2.59 (0.54)	2.85 (0.60)	2.88 (0.52)

Superscript letters refer to pairwise comparisons (conducted for children with AD versus adolescents with AD, children with AD versus non-anxious children, and adolescents with AD versus non-anxious adolescents); means that share subscripts within rows are significantly different at *p* < .05

To address the hypotheses, multivariate analyses of variance (MANOVA), using Pillai’s trace, were carried out, with anxiety (anxiety disorder or non-anxious), age group (child or adolescent) and their interaction entered as independent variables. For the analysis of parenting behavior across the tasks, parental expressed anxiety, intrusiveness, warm engagement and encouragement were entered as dependent variables. Where the effects of the interaction were significant, t-tests were used to explore differences between groups; comparisons were not made between the two non-anxious control groups as this comparison did not relate to the study hypotheses, and to reduce the likelihood of Type I error. To assess age and anxiety group based differences in child/adolescent behavior, MANOVA were conducted with the dependent variable as observed anxiety/avoidance. For child/adolescent observed anxiety/avoidance across the tasks, there was no significant effect of age group (*F*[1, 116] = 0.68, *p* = .41, *ω* ^2^ = < 0.001), but there was a significant effect of anxiety disorder (*F*[1, 116] = 18.10, *p* < .001, *ω* ^2^ = 0.12), with children/adolescents with an anxiety disorder (mean = 1.57, *SD* = 0.36) displaying higher levels of observed anxiety/avoidance than non-anxious children/adolescents (mean = 1.34, *SD* = 0.25). The anxiety disorder x age group interaction was also significant (*F*[1, 116] = 11.16, *p* = .001, *ω* ^2^ = 0.07). Adolescents with an anxiety disorder had significantly higher levels of observed anxiety/avoidance across the tasks, compared to non-anxious adolescents (*t*(58) = 5.11, *p* = .001, *d* = 1.32). There was not, however, a significant difference between children with an anxiety disorder and non-anxious children (*t*(58) = 0.68, *p* = .50, *d* = 0.18), or between children and adolescents with anxiety disorders (*t*(58) = 1.48, *p* = .15, *d* = 0.39). Because there were significant differences between groups in child/adolescent anxiety/avoidance, the analysis of parenting behaviors was repeated using MANCOVA, with child/adolescent anxiety/avoidance entered as a covariate. The results of the MANCOVA were similar to the results of the original analyses and so the original results will be presented but where there were differences between the findings, this will be highlighted. Similarly, although the clinically anxious groups were matched for mood disorder diagnoses, we conducted the analyses examining depressive symptoms, with scores on the SMFQ as a covariate (run separately for parent and child/adolescent report), and then repeated the analyses excluding the five children and five adolescents with comorbid mood disorders. Again, results were broadly consistent but where there was a difference in findings, this is highlighted. Finally, because there were group differences on SES and SDQ-P conduct, further sensitivity analyses were undertaken using MANCOVA, examining parental behavior with SES and then SDQ-P conduct as a covariate. As this did not change the results, analyses are reported without the inclusion of SES or SDQ-P conduct.

Effect sizes were calculated using omega squared (*ω* ^2^) for analyses of variance, with values of 0.01, 0.06 and 0.14 representing small, medium and large effect sizes, respectively (Kirk 1996), and Cohen’s *d* for t-tests, with values of 0.2, 0.5 and 0.8 representing small, medium and large effect sizes, respectively (Cohen 1988).

### Parental Behaviors across the Tasks

There was a significant effect of anxiety disorder (*V* = 0.11, *F*[4, 113] = 3.39, *p* = .01) and age group (*V* = 0.60, *F*[4, 113] = 41.71, *p* < .001) and a significant anxiety disorder by age group interaction (*V* = 0.12, *F*[4, 113] = 3.87, *p* < .01) on parental behavior across the tasks.

**Fig 1:**
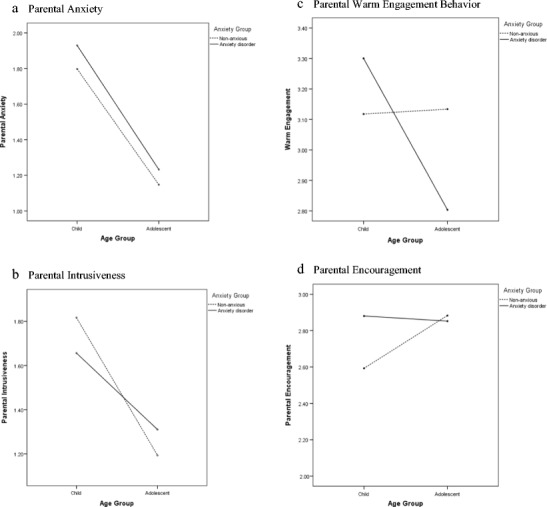
Interactions between anxiety disorder and age group for parental behaviours across tasks

For parental observed anxiety, contrary to our hypotheses, the effect of anxiety disorder did not reach significance, (*F*[1,116] = 2.74, *p* = .10, *ω* ^2^ = 0.01), however the effect of age group was significant (*F*[1,116] = 105.14, *p* < .001, *ω* ^2^ = 0.46), with parents of children (mean = 1.83, *SD* = 0.48) displaying more anxiety than parents of adolescents (mean = 1.19, *SD* = 0.16). As can be seen in Figure 1a, the interaction between age and anxiety group was not statistically significant (*F*[1,116] = 0.13, *p* = .72, *ω* ^2^ = < 0.001).

For parental intrusiveness, contrary to our first hypothesis, there was not a significant effect of anxiety disorder (*F*[1,116] = 0.09, *p* = .76, *ω* ^2^ = < 0.001). However, consistent with our second hypothesis, there was a significant effect of age group (*F*[1,116] = 46.31, *p* < .001, *ω* ^2^ = 0.27), with parents of children (mean = 1.74, *SD* = 0.51) being significantly more intrusive than parents of adolescents (mean = 1.25, *SD* = 0.21). The interaction between age and anxiety groups was also significant (*F*[1,116] = 3.79, *p* = .05, *ω* ^2^ = 0.02). As shown in Figure 1b, parents of adolescents with an anxiety disorder showed significantly higher levels of intrusiveness, compared to parents of non-anxious adolescents (*t*(58) = 2.20, *p* = .04, *d* = 0.58), whereas a similar relationship between parental intrusiveness and anxiety disorder status was not observed among parents of children (*t*(58) = −1.22, *p* = .24, *d* = 0.33). When child/adolescent observed anxiety/avoidance on the tasks was entered as a covariate, however, the age x anxiety disorder interaction effect was no longer significant (*F*[1,115] = 2.83, *p* = .10, *ω* ^2^ = 0.01). The significant interaction effect was also no longer significant when the children and adolescents with a comorbid mood disorder were excluded from the analysis (*F*[1,110] = 1.72, *p* = .19, *ω* ^2^ = < 0.001), or when child-reported (as opposed to parent-reported) SMFQ scores were entered as a covariate ((*F*[1,112] = 2.98, *p* = .09, *ω* ^2^ = 0.01).

Unexpectedly, for parental warm engagement behavior across the tasks there was not a significant effect of child/adolescent anxiety disorder, (*F*[1,116] = 0.92, *p* = .34, *ω* ^2^ = < 0.001). However, in line with our second hypothesis, the effect of age group was significant (*F*[1,116] = 9.71, *p* < .01, *ω* ^2^ = 0.06), and there was a significant effect of the age by anxiety group interaction (*F*[1,116] = 11.04, *p* = .001, *ω* ^2^ = 0.07). As shown in Figure 1c, parents of adolescents with an anxiety disorder showed significantly lower levels of warm engagement behavior, compared to parents of non-anxious adolescents (*t*(58) = −3.44, *p* = .001, *d* = 0.92), whereas a similar relationship between parental intrusiveness and anxiety disorder status was not observed among parents of children (*t*(58) = 1.51, *p* = .14, *d* = 0.39).

Finally, for parental encouragement, contrary to expectation, there was not a significant effect of child/adolescent anxiety disorder (*F*[1,116] = 1.64, *p* = .20, *ω* ^2^ = 0.01), age group (*F*[1,116] = 1.71, *p* = .19, *ω* ^2^ = 0.01), or their interaction (*F*[1,116] = 2.53, *p* = .12, *ω* ^2^ = 0.01) (Figure 1d). When controlling for observed child anxiety/avoidance, however, the interaction effect became significant, reflecting a pattern in which parents of children with anxiety disorders were significantly more encouraging than parents of non-anxious children (*t*(58) = 2.08, *p* = .05, *d* = 0.54), whereas a similar relationship between parental encouragement and anxiety disorder status was not observed among parents of adolescents (*t*(58) = −0.21, *p* = .81, *d* = 0.05).

## Discussion

Parental behaviors, most notably overcontrol, lack of warmth and expressed anxiety, have been implicated in models of the development and maintenance of anxiety disorders in children and young people (e.g., Creswell et al. 2011; Rapee et al. 2009). However, the parenting characteristics of children and young people at different stages of development have not been clearly characterized and consequently, are poorly understood. We examined the parenting behaviors of children with an anxiety disorder, non-anxious children, adolescents with an anxiety disorder and non-anxious adolescents.

As hypothesized and consistent with other studies (e.g., Hudson and Rapee 2001; Verhoeven et al. 2012), we found that parents of children showed significantly higher levels of intrusiveness than parents of adolescents, with an effect size in the large range. Parents of children were also observed to be significantly more anxious than parents of adolescents, with a large effect size, despite there being no significant differences in observed anxiety between the children and adolescents during the tasks. It is possible that parents of children perceive their offspring to be less competent than parents of adolescents in terms of the demands of the tasks (e.g., writing clearly and quickly, generating ideas, planning and organization), and their comparatively higher levels of anxiety and intrusive behavior are a reflection of this. In contrast, it is likely that parents of adolescents recognize that their involvement may be unnecessary given their adolescent’s level of skills/competency to do the tasks, and second, that their involvement may be developmentally inappropriate and may be rejected by their son/daughter.

Consistent with our hypothesis, we also found that parents of children showed significantly higher levels of warm engagement than parents of adolescents, with a small effect. Although these findings differ to those of Hudson and Rapee (2001), this may reflect differences in coding schemes. Specifically, we separated out subdimensions of expressed anxiety, negative and controlling parental behaviors. Our finding that parental expressed anxiety was significantly higher, but parental warm engagement was significantly lower, in children than adolescents may not have been detected had these dimensions been combined together. The finding in relation to warm engagement is consistent with the suggestion that middle childhood is characterized by parent–child relationships that are less challenging than in adolescence (Collins et al. 2002). Possible reasons for this include parenting responses having been influenced by greater levels of general negative affect among their adolescent offspring, less affection towards them as parents, parents no longer being idealized and adolescents and parents spending less time together (Eberly and Montemayor 1999; Larson et al. 2002; Larson and Richards 1991; Laursen et al. 1998; Steinberg and Silk 2002). In addition the parents of adolescents may have been more likely to attribute their offspring’s behaviors to their personality or factors under their control than parents of offspring in middle childhood (who are more likely to attribute children’s behavior to situational pressures or developmental limitations in the child’s knowledge) which lead to higher levels of negative affect in response to offspring behaviors (Dix et al. 1986). Notably, however, in our study there were no significant differences between children and adolescents in the specific dimension of parental encouragement.

Contrary to our hypotheses and the existing literature more broadly (see McLeod et al. 2007; van der Bruggen et al. 2008), we did not find an overall significant effect of child/adolescent anxiety status for any parental behaviors. The finding that child/adolescent anxiety status on its own is not associated with any parenting behaviors is of particular interest given that offspring with anxiety disorders were observed to be significantly more anxious and avoidant than non-anxious offspring during the tasks. This suggests that differences in parental behavior cannot be accounted for by the child/adolescent’s anxiety status alone, and must be seen in the context of the child or adolescent’s age. This is emphasized by our findings that associations between parenting behaviors and anxiety status were moderated by offspring age for parental intrusiveness and warm engagement (and for parental encouragement when controlling for child anxiety/avoidance). Specifically, parents of adolescents with anxiety disorders showed higher intrusiveness and lower warm engagement than parents of non-anxious adolescents, whereas the relationship between these parenting behaviors and anxiety status was not observed among parents of children. The difference in findings between parents of adolescents with an anxiety disorder and non-anxious adolescents is consistent with the existing literature that shows significant associations between adolescent anxiety and perceived parental intrusiveness and lack of warmth (see Waite et al. 2014). Both these parenting dimensions have been associated with psychological control, which may be a particularly relevant construct in relation to adolescence (Barber 1996; Silk et al. 2003). Interestingly, the existing literature shows more consistent findings for the association between adolescent anxiety and perceived parental intrusiveness than parental lack of warmth, whereas we found a large effect size for parental lack of warmth compared to a medium effect for intrusiveness. These differences, and the larger effect sizes found overall, are likely to reflect the use of observation, rather than self-report methodology, as well as the use of a clinical sample (McLeod et al. 2007). Notably in this study parenting behaviors did not differ significantly on the basis of anxiety status.

One possible explanation for the interaction effects might be that parental behavior is merely a reflection of greater anxiety during the tasks among the adolescents with anxiety disorders, however the significant interaction effect for parental warm engagement remained, even when controlling for observed child/adolescent anxiety and avoidance during the tasks and the fact that all groups of children/adolescents exhibited mild levels of observable anxiety across the tasks suggests that this explanation is unlikely. Instead, it appears more likely that parents of adolescents with anxiety disorders, specifically, may step in and be more intrusive in order to protect their child from distress or failure. In addition, the significantly lower level of warm engagement shown by parents of adolescents with an anxiety disorder, compared to those without, may reflect the nature of the parent-adolescent relationship when placed under specific stressors; or perhaps the relationship more broadly, if the adolescent’s anxiety and associated difficulties have resulted in higher levels of frustration and conflict within the family. The lack of association between parental encouragement, which maps most closely onto the construct of autonomy promotion, and anxiety status for parents of adolescents is consistent with research suggesting that psychological control and autonomy promotion are best conceptualized as distinct constructs (Silk et al. 2003) and underlines the importance of disaggregating parenting dimensions that have previously been grouped together.

It is of interest that in this study, the differences between the two child groups only reached significance for parental encouragement. In contrast to the findings with adolescents, parents of children with anxiety disorders appear to show a general pattern of responding to children with anxiety disorders with warmth and (non-intrusive) encouragement. The fact that parents are typically responding in the ways advocated in family based treatments may help explain why family treatments focused specifically on changing parenting behaviors do not necessarily add significant benefits in terms of treatment outcomes for children with anxiety disorders in the study age range (e.g., Reynolds et al. 2012). This is not to say that parents should not be involved in treatment. There is a good deal of evidence demonstrating the effectiveness of parent-focused approaches, especially among younger children (e.g., Cartwright-Hatton et al. 2011; Donovan and March 2014; Waters et al. 2009). However, it may be that a focus on specific parenting behaviors, such as intrusiveness, is unwarranted among children in middle childhood. Having said this, it is important to note that our findings with children are not consistent with a recent report that found significant associations between maternal reported child anxiety symptoms and observations of maternal intrusiveness in children in grades 1, 3 and 5 (6–11 years) (Cooper-Vince et al. 2014). In that study, the association between maternal intrusiveness and child anxiety symptoms was moderated by family financial means; whether our failure to replicate this finding reflects the relatively high economic status of our sample, the inclusion of a clinical (rather than community) population, or differences in the measurement of child anxiety remain unclear. Nonetheless, what is most clear from these findings is the difficulty in drawing conclusions from studies which assess parenting behaviors in the context of offspring anxiety across large age ranges and our findings may, to some extent, explain inconsistent findings across studies (e.g., McLeod et al. 2007).

Limitations to the study should be noted. The children with anxiety disorders were selected to match the adolescents with anxiety disorders on the basis of their primary anxiety disorder diagnosis and comorbid mood and behavior disorders, however, we still cannot be certain that these results cannot be accounted for by other overlapping symptoms, rather than anxiety. As a result of matching the groups for disorders, there are fewer children with a primary diagnosis of separation anxiety disorder than would typically be seen in a general clinic population (Waite and Creswell 2014). We included a range of tasks in order to present scenarios likely to create some mild stress for children and adolescents with a range of anxiety disorders, however it is possible that there may be anxiety-disorder specific associations with particular parenting behaviors in particular contexts (e.g. Wood 2006). We chose the tasks to be mildly stressful and they did invoke mild anxiety for all groups of participants; nevertheless, they may have been differentially demanding for children and adolescents at different developmental levels. Furthermore, the findings may not generalize to situations that invoke greater levels of fear. Indeed, there is evidence to suggest that parental intrusiveness and overprotection are more likely to occur in the context of negative child emotions (e.g., Hudson et al. 2008) and therefore it is possible that our findings would differ with higher levels of child/adolescent negative affect (but for ethical reasons, this would be difficult to test experimentally). Similarly, the artificial nature of the laboratory may mean that the behavior of both parents and their offspring is not generalizable to everyday life and some parental behaviors, such as promotion of avoidance and passivity, may occur in everyday life but occur less frequently in laboratory-based tasks. The different parenting constructs in this study were informed by the wider literature (McLeod et al. 2007; van der Bruggen et al. 2008), however, they may not have captured all relevant aspects of parental over-control and therefore in future research, greater alignment between distinct theoretical constructs and coded behaviors is likely to be beneficial. The cross-sectional nature of this study means that the direction of effects cannot be established and further experimental research is necessary to clarify whether parental behaviors maintain or are simply a response to offspring anxiety disorders. This study considered age within two categories, on the basis that childhood and adolescence can be seen as distinct, developmental periods (Erikson 1968); of course, changes are unlikely to occur in such a discrete way, and future studies should aim to look at still narrower age bands. In addition, families with young children (<7 years) were not included in the present study, and it is during this developmental period that many child anxiety disorders begin, and as such, parenting behaviors may be of particular relevance. Finally, participants were from predominantly White British, relatively high socio-economic backgrounds, and parents were mainly mothers. Future research with more diverse backgrounds, examining parental gender is clearly required, as is identifying other moderating factors, such as the role of child/adolescent gender.

In summary, the findings from the current study suggest that theoretical accounts of the role of parental behaviors in anxiety disorders in children and adolescents should distinguish between these different developmental periods. Although the findings would seem to suggest that a focus on increasing parental warmth and engagement and decreasing parental intrusiveness may be indicated for adolescents, the cross-sectional design of the study means that we cannot be clear about the nature of the relationship between parenting and adolescent anxiety. If the relationship is bi-directional, or if negative parenting behavior results from adolescent symptomatology, as might be suggested by recent prospective studies (Hale et al. 2013; Van Zalk and Kerr 2011; Wijsbroek et al. 2011), then treating the adolescent’s anxiety disorder may actually have a positive effect on parenting behaviors without a specific parenting intervention. Further experimental research to establish causality would be required before committing additional resources to targeting parenting factors within treatment.
